# Numerical analysis of Phase change material and graphene-based tunable refractive index sensor for infrared frequency spectrum

**DOI:** 10.1038/s41598-023-34859-5

**Published:** 2023-05-11

**Authors:** Khaled Aliqab, Kavan Dave, Vishal Sorathiya, Meshari Alsharari, Ammar Armghan

**Affiliations:** 1 Department of Electrical Engineering. College of Engineering, Jouf University, Sakaka, 72388 Saudi Arabia; 2 Department of Information and Communication Technology, Marwadi University, Rajkot, India; 3 Faculty of Engineering and Technology, Parul Institute of Engineering and Technology, Parul University, Waghodia Road, Vadodara, 391 760 Gujarat India

**Keywords:** Engineering, Materials science, Optics and photonics

## Abstract

Here, we present the findings of parametric analysis into a phase transition material Ge2Sb2Te5(GST)-based, graphene-based, with a wide dynamic range in the infrared and visible electromagnetic spectrum. The suggested structure is studied in multi-layered configurations, built up with layers of GST, graphene, silicon, and silver materials. These multilayer structures' reflectance behavior has been described for refractive indices between 1.3 and 2.5. The complete design is simulated using a computational process called the finite element method. Additionally, we have investigated the impact of material heights on the structure's performance in general. We have presented several resonating tracing curves in polynomial equations to determine the sensing behavior across a specific wavelength range and refractive index values. The proposed design is also investigated at various inclined angles of incidence to ascertain its wide-angle stability. A computational study of the proposed structure can assist in the evolution of biosensors to identify a wide range of biomolecules, including malignant, hemoglobin urine, saliva-cortisol, and glucose.

## Introduction

The integration of life sciences and electronics has produced a powerful resource for studying and measuring bio-molecular interactions. Over the past several years, electronic devices have significantly contributed to the characterization and analysis of bio-atomic interactions in life science^1^. Interest in these electronic devices has surged in several fields, including but not limited to synthetic identification, genomics, clinical detection, and proteomics^2,3^. The drug research, biomedical, food safety, defense, security, and environmental monitoring fields have all realized the critical relevance of biosensor use. As a result, scientists have developed sensitive analytical techniques based on biosensors that can detect minute changes in biological samples with great precision. Biosensors are diagnostic devices that use a biological detecting component and have many practical uses in fields as diverse as drug development, medical diagnosis, food processing, environmental monitoring, military defense, and national security^4^. The first biosensor, which employed an immobilized glucose oxidase electrode to detect oxygen or hydrogen peroxide electrochemically, was developed by Clark and Lyons to quantify glucose in biological samples^5^. Since then, biosensor technology and applications have advanced tremendously because of novel techniques in fields ranging from electrochemistry and nanotechnology to bioelectronics^6^. An optical biosensor is essentially a bio-recognition element within a short distance of a hardware transducer, which converts the capture of an analyte into a detectable shift in some aspect of light's properties (such as its intensity, wavelength, resonance, or refractive index). Interferometers^7^, gratings^8^, plasmonics^9^, and resonators^10^ are only a few examples of physical transduction mechanisms that may be utilized in optical sensing. Regarding sensors, those based on plasmonics are perhaps the most well-known and commonly used^11^. To many, the Surface Plasmon Resonance (SPR) biosensor represents the pinnacle of optical and plasmonic biosensor technology^9^. The first recorded evidence of SPR occurred in the physical world in 1902. This obscure optical phenomena observation developed over decades into a solid insight into surface plasmon physics^12^. Liedeberg and Nylander first proved surface plasmon resonance (SPR) as a useful optical biosensor in 1982 for gas detection and bio-sensing^13^. Since then, SPR has fortified surface chemistry by serving as a gateway where chemistry, physics, and biology may all converge^14^. As the prospects for surface plasmon resonance (SPR) based biosensors continue to expand rapidly^15^, there has been a recent explosion in the number of researchers interested in the topic and the SPR technique has gained traction in biosensors as a means of detecting^16^. Due to their beneficial qualities, such as their capability of continuous detection on a label-free system, constant observation, prompt reaction, and heightened sensitivity, as well as their noteworthy advantages like design flexibility, miniaturization, multiplexing of sensing data, and remote sensing^17^, SPR technology has broadened its potential application areas from the biomedical to the environmental and even the industrial. Successful commercialization and widespread use of SPR-based biosensors for detecting a wide variety of biomolecules, including nucleic acid, proteins, a plethora of enzymes, growth factors, DNA, antibodies, medicines, and food quality, have been achieved in recent years^18,19^ but before everything else, SPR's biomedical applications are especially groundbreaking^20^. Collective electron oscillations in metals are called plasmons, and they can be either propagating surface plasmons (PSPs) that travel along metal-dielectric interfaces or localised surface plasmons (LSPs) that are confined to the surface of a metallic nanostructure (with dimensions smaller than the wavelength of light) (LSPs). It is a crucial tool for probing surface processes because the coupling of these modes to incoming light results in resonances that rely heavily on the compositions, shapes, and sizes of the metal nanostructure and the dielectric characteristics of the surrounding medium. Both SPs and LSPs have an electromagnetic field that is localised at the surface and decays exponentially into the ambient medium with half-lives of 30 nm and 200 nm, respectively. As a result, sensors built on these processes are highly attuned to changes occurring close to the ground. Physicochemical contact with the analyte causes a change in the refractive index of the sensing layer around the metallic nanostructure, which is the basis for SPR and LSPR sensors^21^.

Recent advancements in 2D materials have tremendously improved the optical performance of SPR biosensors. Efforts have been made before to strengthen the electromagnetic field when SPPs are excited at the metal-dielectric surface by using the metal periodic grating structure. Diffraction in a metal grating in a prism-based SPR biosensor can increase the electromagnetic field in the vicinity of the metal surface. When light is incident on a metal grating, it interacts with the periodic structure of the grating and creates diffraction patterns, which cause the light to be diffracted in different directions. This diffraction can create regions of constructive and destructive interference of the light waves, leading to an increase or decrease in the electromagnetic field strength^22^. Dispersion engineering is an important technique for improving the performance of SPR sensors by using plasmonic nanostructures, such as nanoparticles, nanorods, or nanowires. These structures can support localized surface plasmon resonances, which can be tuned by changing their size, shape, and composition. The plasmon resonance can also be coupled to the SPR resonance, leading to enhanced sensitivity and spectral selectivity^23,24^. Multilayer structures can also be designed to manipulate the dispersion of light in an SPR setup by controlling the effective refractive index of the structure. By carefully selecting the thicknesses and refractive indices of each layer, the phase velocity of light can be adjusted, which in turn affects the resonance condition for SPR^25^. This enables precise tuning of the sensor's sensitivity and spectral response^26,27^ gave the idea about enhanced sensitivity using nanogratings and dispersion engineering in SPR theoretically^28^ proved this fact experimentally as well which proved to be the main inspiration for us^29^ helped us in selecting the layer in multilayer structure for dispersion engineering^30^ aided us in merging two famous topics for improvement of SPR biosensor i.e. semi-conductor and 2D material (graphene sandwiched between Si)^31–33^ helped to solve the problem of tuning and sensitivity in the infrared region. The novelty of the proposed SPR biosensor to the best of the author’s knowledge is in combining metallic nano-grating periodic structures with sandwiched multilayers of graphene and GST material between Si as a dielectric substrate.

## SPR principle and selection of materials in the proposed biosensor

Now, we have to think about how to excite the surface plasmons (SPs); light waves excite the surface plasmons in optical sensors based on surface plasmon resonance (SPR). The phase-matching criterion for surface plasmons’ optical excitation states that the projection along the x-axis of the input light wavevector must equate to the constant of propagation of surface plasmons i.e. kSP. There are generally three methods: prism coupling, slot waveguide, and v-groove waveguide^22^; generally, prism is preferred^16^. In prism coupling, surface plasmons can only be optically excited by boosting the wave vector of the incident light. The attenuated total reflection (ATR) approach is achieved by directing the light wave through a medium with a higher optical density^34^. To use this prism method for achieving SPR, there are two geometrical configurations, possibly Otto and Kretschmann configurations, and out of that Kretschmann is preferable^16^. The Kretschmann configuration is achieved by evaporating the metal film onto a prism or other high-index glass block. The prism is illuminated, and a fleeting wave of light travels through the metal film. Between two different RI media, one with a lower RI (like water) and one with a higher RI (like air), a thin metal film is created, where the plasmons are stimulated. Most commercial SPR devices employ the Kretschmann setup, where ligand molecules are immobilised on a metal surface and addressed by molecules of analyte in a mobile phase. SPR angle shifts if binding to the immobilised ligand alters the local effective refractive index. This may be tracked in real-time, with a sensorgram being generated as evidence. The amount of mass collected by individual immobilised ligand molecules may be calculated from the magnitude of the resulting change in the SPR signal. The Kretschmann arrangement allowed for more creative leeway in the liquid handling system's layout. Light from the medium with the higher refractive index (the prism) does not penetrate the liquid but is instead reflected to the sensor surface, which is coated with a thin metal coating.

By measuring the shift in RI of an analyte caused by biomolecule interactions with the sensor, SPR sensors operate. The SPR condition is set by the degree to which the evanescent wave produced by the TM light and the SP wave are in phase with one another. When this occurs, a shift in the reflectance profile can be observed. Several factors, such as the prism adopted, the wavelength of the incident light, the nature of the 2D material, the metal type, and the biomolecule to which it is bound, determine the precise angular location of the reflectance dip. Out of this, metal plays a significant role in the SPR phenomenon; generally, Copper, Aluminum, Silver, and Gold are used. Silver and gold are the best possible options for SPR-based biosensors. The high sensitivity that results from the resonance angle change that gold provides owing to variations in the refractive index of the sensing medium makes it a desirable metal for use in the sensor^35^. Moreover, gold is a chemically inert substance that exhibits stability in the air. However, an silver metal layer base sensor is more precise than a gold one, showing a sharper resonance dip with improved clarity and sharpness and narrower full width at half maximum (FWHM) compared to gold^36^. However, silver demonstrates poor stability because of its susceptibility to oxidation in the presence of sensing material, and as a result, the sensor's sensitivity drops as the silver layer oxidizes. To prevent silver from oxidizing, several long-lasting metallic or dielectric coatings have been proposed^37^. At the interface between the dielectric and the analyte, the field strength of the excited light may be amplified by placing a dielectric layer with a high refractive index, such as silicon, onto an SPR active metal, such as silver^38^. When a silicon layer is present, the metal and silicon layers both participate in absorption^39^. Because of this enhanced absorption, the dielectric contact experiences a greater rise in field strength. As a result, there is increased stimulation of SPs. For this reason, silicon is a frequent part of today's biosensors to increase sensitivity and stability^40,41^. The ability of the sensor surface to adsorb the analyte is a good measure of the SPR sensor's performance. Two-dimensional (2D) nanomaterials like graphene (G), and transition-metal dichalcogenides (TMDGs), black phosphorus (BP), have attracted a lot of attention as potential components of SPR sensors because of their unusual electrical, optical, and catalytic capabilities and have found utility in cutting-edge biosensing technologies^42^. Graphene is the most prominent material of this kind. Graphene's hexagonal cells engage through pi-stacking interactions with the ring structures made of carbon often seen in biomolecules, causing strong and stable adsorption of the biomolecules, carbon atoms in a hexagonal lattice form graphene, a two-dimensional material with extraordinary properties which is the thinnest artificial planner material. Graphene has exceptional mechanical, optical, and electrical characteristics^43^. Due to its pi -stacking structure, it is particularly useful for detecting aromatic chemicals and exhibits excellent tenability, low loss, high captivity, a large surface-to-volume ratio, high electron mobility, high optical transparency, and improved ability to contact the analyte's molecule^44^. Therefore, adsorbates readily interact with this structure, elevating adsorption levels that are amenable to use in biosensors^45^. However, the important factor to note here is that the graphene is not directly in contact with the analyte because it could be easily wrecked and therefore sandwiched between two silicon substrates. Silicon provides stability and mechanical support. Additionally, in several papers, it has been found that the usage of Silicon helps in improving the sensitivity of the biosensor^2,29,46,47^. Silver rectangular gratings can be used in Surface Plasmon Resonance (SPR) biosensors to increase the adsorption of the analyte. The grooves in the grating provide additional surface area for the analyte to adsorb onto, which can increase the sensitivity of the biosensor.

Additionally, the grooves in the grating can act as a physical barrier to prevent non-specific binding of the analyte, increasing the biosensor's specificity. When incident light is shone on the grating at a specific angle, it can excite a Surface Plasmon or localized plasmon resonance if it’s a small metal nano particle^48^, a collective oscillation of free electrons in the metal, which results in an enhancement of the electromagnetic field in the vicinity of the metal surface. This electromagnetic field enhancement can increase interaction between the analyte and the biosensor, thus increasing adsorption^49^. Metal nanograting, used as a surface relief pattern, has been frequently used to further enhance the performance of SPR sensors based on the Kretschmann arrangement. It is important to keep in mind that the presence of a metallic grating can boost the sensor's performance due to localised surface plasmon (LSP) resonance with highly amplified field intensity and sensitivity augmentation by an increase in the surface reaction area, which mediates extra couplings among stimulated plasmons and local binding events^50^. Nanograting surface plasmon resonance (SPR) sensors have a straightforward architecture, making them amenable to industrial production. Recent advances in nanolithography and nanofabrication techniques may allow for the mass production of these sensor devices at a reasonable cost. As a result, nanograting SPR sensors can be a cost-effective resource for high-throughput screening and have been researched thoroughly^51–57^

Silicon and Graphene layers are added for better sensitivity and adsorption. Still, tuning is also a crucial factor to consider in biosensor design, and phase-changing materials are a viable option for the same. Light's phase is an essential characteristic. Applications in holography beam steering, frequency modulation, sensing, and other fields benefit significantly from the capacity to modify the wavefront, which is made possible by controlling the phase. The ability to swiftly and substantially modify PCMs (Phase Changing Material)' optical and electrical characteristics lays the groundwork for developing possibilities of PCMs in photonics. The phase-changing material Ge2Sb2Te5 (GST) has two forms: amorphous and crystalline. The phase-changing material may be transformed into these two forms by electronic, thermal, or optical stimulation. The electrical and optical characteristics of the two forms are distinct. Changing the GST phase in the local region would allow for adjusting the polariton mode's effective index. This is because the material has various refractive indices in different forms. These varied characteristics allow for spectral fine-tuning. GST phase transition materials have a higher absorption rate in the visible and near-infrared spectrum over other 2D materials like graphene, MoS2, and WS2. The crystalline form of GST is extremely absorptive even below the bandgap, resulting in a considerable absorption of light, and prism coupling relies heavily on shifting the resonance frequency of the transmission or reflection spectra. The evanescent coupling to the PCM is sensitive to the real and imaginary parts of the refractive index, generally indicated as n and k, which changes whenever the solid phase is altered. Therefore, a situation near to zero-reflection may be attained by meticulously optimizing the thickness of the GST layer. This near-zero reflection phenomenon is characterized by a fast dimming of reflected light and a noticeable phase change at the resonance angle, both of which can be utilized to significantly boost sensitivity utilizing plasmon resonances.

Temperature-controlled aGST and cGST SPR biosensors use a phase change GST material and are controlled by temperature. The two types of GST commonly used in these biosensors are aGST and cGST. aGST is an amorphous form of GST with a lower melting point than c-GST, which is the crystalline form. Because of its lower melting point, aGST can easily switch between its solid and liquid phases by controlling the temperature. This can be used to fine-tune the sensitivity of the biosensor to the target molecule. cGST, on the other hand, has a higher melting point than aGST and is more stable in its solid phase. It can increase the specificity of the biosensor, as well as its long-term stability. Additionally, cGST can be functionalized with a wide variety of biomolecules, such as antibodies or enzymes, to increase the specificity of the biosensor for a particular target molecule.

By using temperature-controlled aGST and cGST SPR biosensors, researchers can optimize and control the sensitivity and specificity of the biosensor to the target molecule. The ability to switch between aGST and c-GST can also provide a method for multiplexing the biosensor, enabling the detection of multiple target molecules in a single sample.

## Theoretical modeling and design considerations

**Figure 1:**
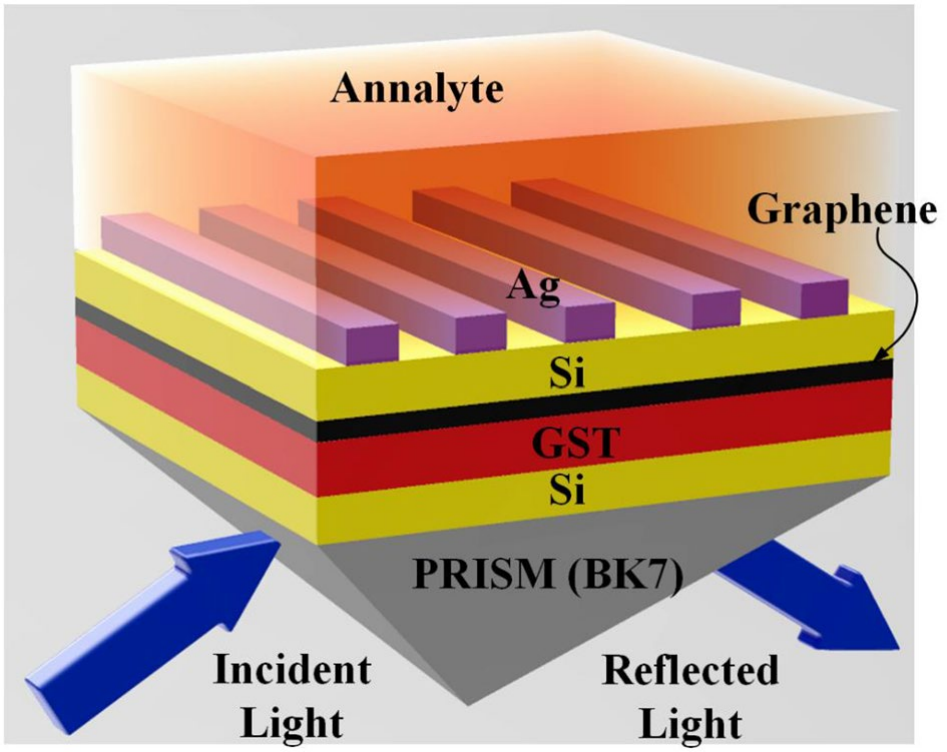
Three-dimensional view of the proposed multi-layered SPR biosensor.

Figure 1 depicts the proposed modified SPR biosensor in a typical Kretschmann configuration. The theoretical setup consists of the laser source, prism, and photodetector. The incident TM polarized light is exposed to the surface of the BK7 prism and undergoes total internal reflection inside the prism and then the photodetector captures and analyzes the refracted optical signal at the other end. Unlike the conventional SPR sensor (Prism-Metal-Dielectric-Analyte), the proposed sensor is a multilayer structure with added layers such as graphene and GST for sensitivity and tuning enhancement. The theoretical model comprises 7 layers (BK7–Si–GST–Graphene–Si–Silver–Analyte) whose source is 1.3 to 2.5 um for the computations and its electrical field distribution and modeling is done in COMSOL multiphysics software. All the layers are piled up in the vertically upright position to the prism forming a symmetrical pyramid and are defined by their respective thickness, real and complex refractive indices, and dielectric constants. The recommended design utilizes a coupling prism, which is BK7 and due to its low refractive index, the BK7 prism is the best option. The use of a low refractive index prism to boost biosensor sensitivity and functionality has recently received much attention. When comparing the resonance curves of high- and low-RI prisms, it is clear that the former produces a more substantial dip. The angle of resonance, sensitivity, a shift in the resonance curve, and FWHM values obtained from a low refractive index prism are superior to those obtained from a high refractive index prism^58^. This can be demonstrated mathematically in the case of a p-polarized light incident on the prism and the evanescent wave produced as a result of the prism's absorption, transmission, and reflection.

### Resonance condition

When an incoming light causes free electrons to pair with a metal surface that is in contact with a dielectric, the electric field has a sharp break in amplitude in the direction of the normal to the surface. Since the E component of s-polarized (TE mode) waves is orthogonal to the surface normal, they cannot sustain surface plasmons, which are p-polarized (TM mode) in nature. Electromagnetic components of incident light with p-polarization are shown in Eqs. (1–2).

1 $${\overrightarrow{E}}_{i}=\left({E}_{{i}_{x}},0,{E}_{{i}_{z}}\right){e}^{i\left({k}_{{i}_{x}}x+{k}_{{i}_{z}}z-\omega t\right)}\left[\frac{\mathrm{V}}{\mathrm{m}}\right]$$

2 $${\overrightarrow{H}}_{i}=\left(0,{H}_{{i}_{y}},0\right){e}^{i\left({k}_{{i}_{x}}x+{k}_{{i}_{z}}z-\omega t\right)}[\mathrm{A}/\mathrm{m}]$$

On applying appropriate boundary conditions after introducing the above equations into Maxwell’s equations, we can get the equation for achieving resonance, which is as follows in Eq. (3):

3 $$\frac{2\pi }{\lambda }\sqrt{{\varepsilon }_{p}}\mathrm{sin}{\theta }_{RES}=\frac{\omega }{c}\sqrt{\frac{{\varepsilon }_{m}{\varepsilon }_{a}}{{\varepsilon }_{m}+{\varepsilon }_{a}}}$$

Here, the light speed is c, λ is the wavelength of the incident light, ώ is the angular frequency, θ_RES_ is the incident angle, ${\varepsilon }_{p}$ is the prism’s permittivity, ${\varepsilon }_{m}$ is the permittivity of the metal and ${\varepsilon }_{a}$ is the adjacent medium’s permittivity. Above equation can be further simplified to Eq. (4)^34^.

4 $${k}_{x}=\frac{2\pi }{{\lambda }_{0}}{n}_{p}\mathrm{sin}\theta =\mathrm{Re}\left\{{k}_{SP}\right\}$$

where k_x_ is the x-direction wave vector, n_p_ is the prism’s refractive index, θ is the incident angle, λ_0_ is the vacuum’s wavelength and $\mathrm{Re}\left\{{k}_{SP}\right\}$ defines the real part of the SP wave vector in the x-direction at the boundary between metal and dielectric.

### Refractive index of multilayers in proposed biosensor

We have numerically investigated a variety of SPR biosensor configurations to see which ones gave the most effective results. For both the forms of GST i.e., crystalline and amorphous, authors simulated the proposed multilayer structure without graphene, and then the graphene was added to both structures. After that, various simulations were performed to get the most optimized version of each layer. The first layer in all these structures is always the BK7 prism layer and the refractive index of the BK7 prism for the wavelength range of 0.3 to 2.5 um is given by Eq. (5). The refractive indices of PCM, such as aGST and cGST were calculated as the function of frequency. The real part of aGST is in the range of 2.6 to 4.6 and the imaginary part is in the range of 0 to 2.4 for the range of 100 to 800 THz. Similarly, the real part of cGST is in the range of 2.25 to 7.16 and the imaginary part is in the range of 0 to 4.1 for the range of 100 to 800 THz. In the COMSOL, by using the finite element method the effect of the graphene layer was analyzed for both the phase-changing material aGST and cGST by plotting the SPR curve to look for the resonance shift. At first, both the structure were simulated without the graphene layer, and the SPR curve was analyzed, after that one layer of 0.3 thickness of graphene was added for the simulation.

**Figure 2:**
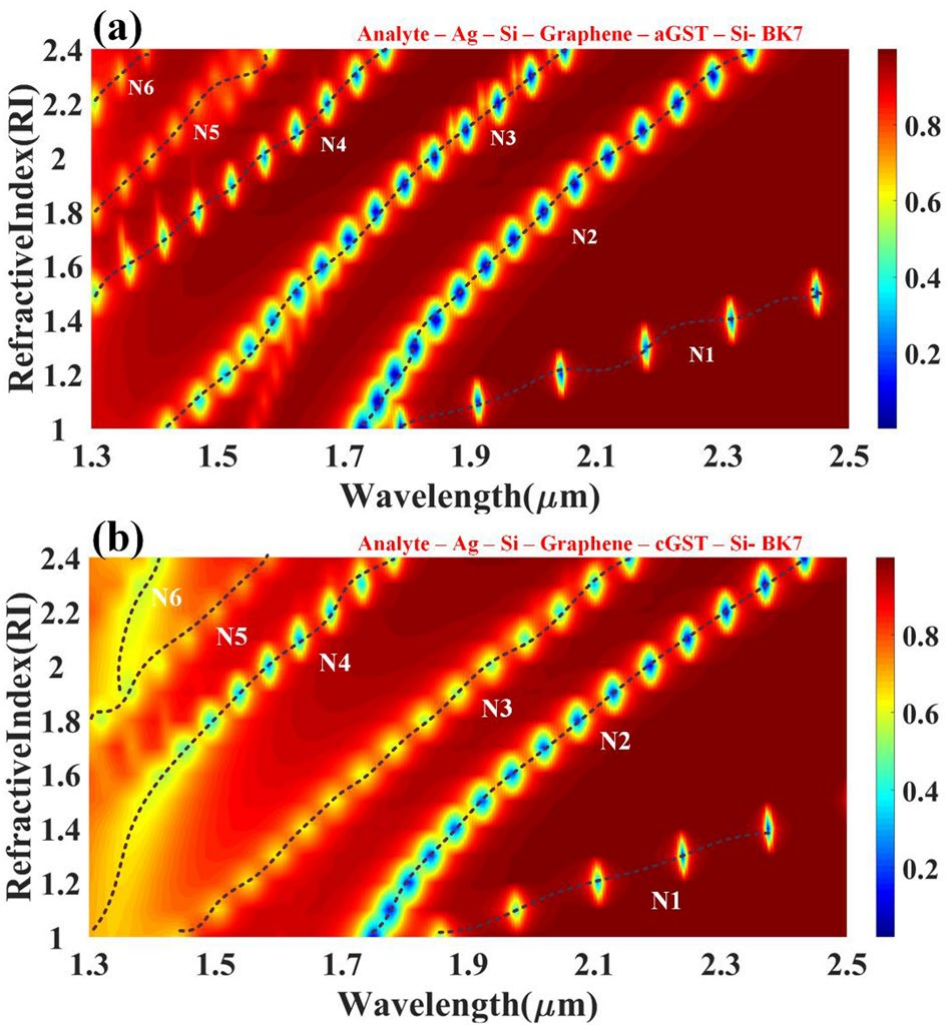
Contour map depicting the modification in resonating peaks for the multi-layered structure's simulated reflectance response as the wavelength function over the range of 1.3 to 2.5 µm w.r.t change in refractive index. Variation in the reflectivity distribution of the (**a**) aGST and (**b**) cGST phase in the structure can be observed with the traces of N1 to N6 resonating peaks for the 1 to 2.4 RI values.

Figure 2 shows the impact of the refractive index on the reflectance for a different phase of the GST material. For both the forms, 6 resonant peaks are observed between 1.3 to 2.5 um wavelength range. These traces have a quadratic relationship between refractive index and RI. Results of aGST are slightly better than cGST for the RI range of 1.8 to 2.4 near 1.3 um. The effect of tunability is also observed because of observant shifts in peaks in almost all of the wavelength range.

5 $${n}_{BK7}=\left(\frac{1.03961212{\lambda }^{2}}{{\lambda }^{2}-0.00600069867}+\frac{0.23179344{\lambda }^{2}}{{\lambda }^{2}-0.0200179144} {\left.+\frac{1.01046945{\lambda }^{2}}{{\lambda }^{2}-103.560653}+1\right)}^{1/2}\right.$$

For optimal performance, graphene is sandwiched between the layer of Silicon whose thickness is 40 nm, and the refractive index of the Silicon is calculated by Sellmeir’s equation as follows Eq. (6):

6 $$\begin{array}{c}\\ {n}^{2}\left(Silicon\right)=1+\frac{10.6684293{\lambda }^{2}}{{\lambda }^{2}-(0.301516485{)}^{2}}+\frac{0.003043475{\lambda }^{2}}{{\lambda }^{2}-(1.13475115{)}^{2}} +\frac{1.54133408{\lambda }^{2}}{{\lambda }^{2}-(1104.0{)}^{2}}\end{array}$$

where λ is incident light’s wavelength in the µm range. Two parameters—plasma wavelength (${\lambda }_{\mathrm{p}}$) and (${\lambda }_{\mathrm{cb}}$) are bulk collision wavelength—can be used to characterize the spectral characteristics of any given piece of bulk metal. Specifically, the plasma wavelength is the wavelength that correlates to the frequency of the metal's electron density oscillations; collisions between electrons in the bulk metal damp electron density oscillations. The corresponding wavelength is called the bulk collision wavelength. Plasma wavelength can be calculated with the following formula shown in Eq. (7).

**Figure 3:**
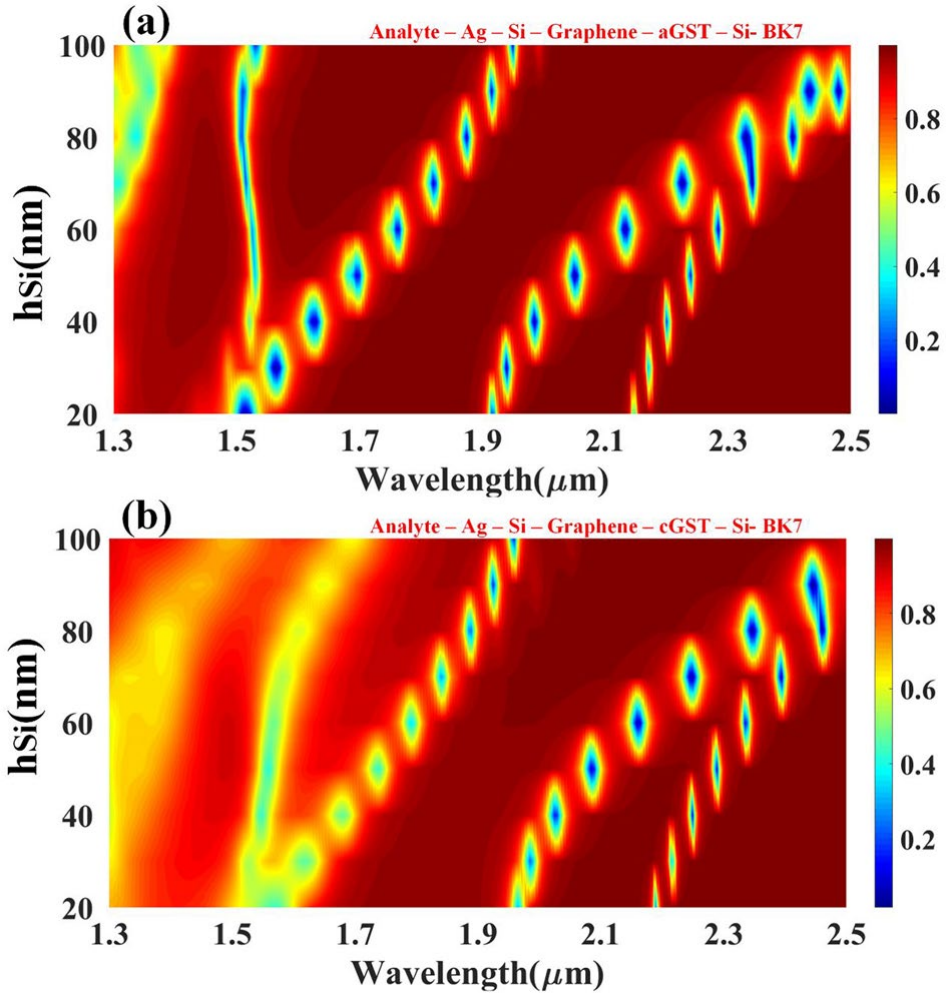
Contour map depicting the modification in resonating peaks for the multi-layered structure's simulated reflectance response as the wavelength function over the range of 1.3 to 2.5 µm w.r.t change in the height of the silicon layer. Variation in the reflectivity distribution of the (**a**) aGST and (**b**) cGST phase of the material.

This proposed design has been flexible and generalized for a wide range of wavelength i.e., 1.3 to 2.5 um, the effect of metal height, width, presence and absence of metamaterials, and phase change materials, the incident angle has been studied and the peak has been observed for different analytes in the given range. For the analyte detection, this generalized SPR works in a way that for a given wavelength and other fixed parameters such as metal height, width, metamaterial, phase change material fixed dimensions, one parametric equation is calculated whose solution will give you the type of analyte which this generalized sensor can discover. All the parameters such as metal height, width, the refractive index of analyte, graphene and GST thickness and height, etc. are kept constant and silicon height is varied from 20 to 100 nm during the simulation to find out the influence of silicon height for resonating peaks for a particular wavelength range. Figure 3 shows the impact of silicon height on the reflectance response with Fig. 3a showing for aGST and Fig. 3b for cGST. It can be observed that numerous resonance peaks occur after 1.5 µm in both phases. Better peaks were perceived in aGST in comparison with cGST because of the refractive index of the aGST phase. The resonance condition keeps on increasing by increasing the height of the silicon without any changes in the reflectivity distribution for that particular wavelength range. The quadratic relationship has been established between the height of the silicon and wavelength for the resonance condition. The solution of the quadratic equation will give you the optimized height of the silicon for that precise wavelength range. The tunability of the structure can be easily spotted because of the obvious shift in the structures by changing the phases.

7 $$\frac{1}{{\lambda }_{\mathrm{p}}}=\sqrt{\frac{N{e}^{2}}{4{\pi }^{2}{c}^{2}m{\varepsilon }_{0}}}$$

Collision wavelength can be calculated by the following formula shown in Eq. (8):

8 $$\frac{1}{{\lambda }_{\mathrm{cb}}}=\frac{{v}_{\mathrm{f}}}{2\pi c{R}_{\text{bulk}}}$$

Here, light speed is $c$, $m$ is the electron’s mass, $e$ is the electron’s charge, $N$ is the concentration of the electron, ${\varepsilon }_{0}$ is the vacuum’s permittivity, ${v}_{\mathrm{f}}$ is the velocity of electrons at Fermi energy and R_bulk_ is conduction electrons' mean free channel at Fermi energy. For the conductivity of the metal, one must consider a simplistic yet realistic model, which provides an optimum result. The Lorentz Drude model, a classical mechanics approach for explaining the electromagnetic properties of metals, is based on several key assumptions for finding conductivity in metals. This approach gives an accurate description of the metals such as gold, silver, or aluminum. For parameterizing optical constants in the metal, the Lorentz-Drude model is the best possible option^59^. Bound and unbound electrons both contribute to the optical characteristics of typical metal-based media. As a result, in the corresponding complex dielectric permittivity, the Drude component for the intraband effect and the Lorentz term for the interband transition are both incorporated in the form of the Drude- Lorentz model^60^. According to the free electron Drude model, the metal's complex dielectric constant may be written as a function of both the wavelengths i.e. plasma and collision using the formula shown in Eq. (9)^61^.

9 $${\varepsilon }_{\mathrm{m}}(\lambda )={\varepsilon }_{\mathrm{mr}}+\mathrm{i}{\varepsilon }_{\mathrm{mi}}=1-\frac{{\lambda }^{2}}{{\lambda }_{\mathrm{p}}^{2}\left(1+\mathrm{i}\frac{\lambda }{{\lambda }_{\mathrm{cb}}}\right)}$$

where λ is the particular wavelength from the targeted wavelength range, λ_p_ is the plasmonic wavelength and λ_cb_ is the collision wavelength. The value of plasmonic and collision wavelength for the appropriate wavelength range and particular silver metal has been taken as 1.4541 × 10^–7^ m and 1.7614 × 10^−5^ m respectively from^62,63^. From this, the refractive index is calculated as Eq. (10).

10 $$\mathrm{n_{Ag}}(\lambda )=\sqrt{1-\frac{{\lambda }^{2}{\lambda }_{c}}{{\lambda }_{p}^{2}\left({\lambda }_{cb}-i\lambda \right)}}$$

For the graphene layer, the RI (ng) is expressed as Eq. (11)^64^

11 $${n}_{\mathrm{g}}=3+\lambda \frac{C}{3}i$$

where C is the constant with the value of 5.446 μm^−1^.

## Numerical analysis in the modeling of the proposed biosensor

There are three approaches, namely the transfer matrix method, the field tracing technique, and, the resultant wave method that may be used to derive an equation for radiative characteristics, such as reflectance and transmittance, of the multilayer, as in the Kretschmann configuration. As there are no approximations in the transfer matrix approach, it is regarded as the most accurate of these techniques^65^. Therefore, to investigate the performance characteristics of the proposed multilayer structure for parallel polarization light entering through a prism such as reflectance, we will be implementing the TMM (Transfer Matrix Method) on the biosensor. Applying boundary conditions, the following matrix equation explains the connection between the electric field and magnetic field components along the tangential direction at the first and final boundary layers shown in Eq. (12)^66^.

12 $$\left[\begin{array}{c}{E}_{1}\\ {H}_{1}\end{array}\right]=T\left[\begin{array}{c}{E}_{N-1}\\ {H}_{N-1}\end{array}\right]$$

where E_1_ and E_N-1_ are electric field components of the 1st and Nth layer respectively, H_1_ and H_N-1_ are magnetic field components of the 1st and Nth layer respectively. T is the characteristic matrix representation for the generalized N layer model and can be further simplified as Eq. (13)^67^

13 $${T}_{ij}={\left(\prod_{m=2}^{N-1} {T}_{m}\right)}_{ij}=\left[\begin{array}{c}{T}_{11} {T}_{12}\\ {T}_{21} {T}_{22}\end{array}\right]for i,j=\mathrm{1,2},\dots$$

Now, the formula shown in Eq. (14)^56^ must be used to calculate every layer's admittance and phase shift to build the transfer matrix.

14 $${\beta }_{m}=\frac{2\pi }{\lambda }{d}_{m}\sqrt{{n}_{m}^{2}-{\left({n}_{p}\mathrm{sin}\left({\theta }_{in}\right)\right)}^{2}},{q}_{m}=\frac{\sqrt{{n}_{m}^{2}-{\left({n}_{p}\mathrm{sin}\left({\theta }_{in}\right)\right)}^{2}}}{{n}_{m}^{2}}$$

where q_m_ is the admittance of layer m and β_m_ is the phase shift of layer m, respectively. For finding these, parameters such as n_m_ which is layer m’s refractive index, d_m_ which is layer m’s thickness, n_p_ which is the prism’s refractive index, and θ_in_ which is the prism’s incident angle must be known. For the N-layer surface plasmon sensor, where various reflections arise at each layer's interface depending on the incoming light at the prism and the first layer, it is necessary to account for the aggregate of these reflections when calculating total reflection. P- polarized propagating wave through the successive layers in the N-layer model can be characterized by the Transfer matrix as shown in Eq. (15).

15 $${T}_{m}=\left[\begin{array}{cc}cos\left({\beta }_{j}\right)& -\mathrm{i sin}\left({\beta }_{j}\right)/{q}_{j}\\ -i{q}_{j}\mathrm{sin}\left({\beta }_{j}\right)& \mathrm{cos}\left({\beta }_{j}\right)\end{array}\right]$$

On further mathematical simplifications, the reflection co-efficient for p-polarized incident light in N-layer proposed biosensor is calculated as Eq. (16).

16 $${r}_{p}={\left|\frac{\left.\langle {T}_{11}+{T}_{12}{q}_{N}\right){q}_{1}-\left({T}_{21}+{T}_{22}{q}_{N}\right)}{\left({T}_{11}+{T}_{12}{q}_{N}\right){q}_{1}+\left({T}_{21}+{T}_{22}{q}_{N}\right)}\right|}^{2}$$

At last, the reflectance of the overall multilayer design is expressed as ${R}_{p}={\left|{r}_{p}\right|}^{2}$

## Optical performance parameters of the proposed biosensor

Analyses of several characteristics, including detection accuracy, sensitivity, resonance angle shift, FoM (Figure Of Merit), full wave half maximum, and quality factor, are used to evaluate any SPR-based biosensor's optical performance. To be called a good and reasonable functioning biosensor, its detection accuracy, figure of merit(FoM), and sensitivity should be, to the highest extent feasible^16,68^. The variation in a shift of the resonance point caused by biomolecular adsorption is what is meant by the concept of sensor sensitivity (S) for SPR sensors. This variation is due to a dimension change in the RI of the medium that is being sensed. In simpler terms, it is the ratio of change in output i.e., resonance wavelength w.r.t. to the change in the refractive index of the medium. It is being computed as follows: its unit in SPR (Eq. 17) biosensor is nm/RIU, as any change in the analyte's RI causes the resonance dip to shift in angular location^69^.

17 $$\begin{array}{c}S=\frac{\Delta {\lambda }_{sp}}{\Delta {n}_{s}}\end{array}$$

where S is angular sensitivity, ${\lambda }_{sp}$ is the shift in the wavelength of resonance, and Δn_s_ is the variation in the index of refraction of the dielectric sample. For a given change in the index of refraction of the SPR sensor, a biosensor's sensitivity grows in direct proportion to the magnitude of the resonance wavelength shift. The working principle of the biosensor is majorly based on the shift of resonance wavelength for the resonance condition with the smallest variation in a refractive index; therefore, high sensitivity will make the sensor effective. Table 1 shows the derived quadratic equation for the different traces generated based on the results of a numerical study of the suggested sensor model. The quadratic equation is identified for both phases of the proposed GST structure (aGST and cGST). It can be observed that the values of each quadratic equation are valid for the specific range of the wavelength and refractive index values. The values of this range and its quadratic equation will help us to choose the sensing material and operating wavelength. The wide range of the wavelength and optical sensing values allows the proposed structure to work for a wide range of biomolecule sensing devices (glucose, hemoglobin, cortisol, urine, etc.) because most of these molecules have refractive index ranges between 1 and 2.5.

**Table 1:** Derived quadratic equation for the respective reflectance traces for the specific range of the refractive index and wavelength range.

Structure type	Quadratic equation	Fitting curve	Refractive index range	Wavelength range
Analyte–Ag–Si–Graphene–aGST–Si–BK7	$\lambda =-0.3125{n}^{2} +2.1812n -0.1225$	N1	1–1.5	1.74–2.45
	$\lambda =0.1371{n}^{2} -0.0226n+1.6119$	N2	1–2.4	1.723–2.35
	$\lambda =0.0615{n}^{2} +0.2309n +1.1386$	N3	1–2.4	1.42–2.05
	$\lambda =-0.0663{n}^{2}+0.7725n +0.2942$	N4	1.5–2.4	1.30–1.76
	$\lambda =0.1310{n}^{2} -0.0929n +1.0487$	N5	1.8–2.4	1.30–1.61
	$\lambda =-0.0000{n}^{2}+0.4000n +0.4250$	N6	2.2–2.4	1.30–1.38
Analyte–Ag–Si–Graphene–cGST–Si–BK7	$\lambda =0.4643{n}^{2}+0.1807n+1.2133$	N1	1–1.4	1.85–2.37
	$\lambda =0.1445{n}^{2}+ 0.0078n+1.5905$	N2	1–2.4	1.74–2.44
	$\lambda =0.0839{n}^{2}+0.2059 +1.1701$	N3	1–2.4	1.45–2.14
	$\lambda =0.2165{n}^{2}-0.3885n + 1.4674$	N4	1–2.4	1.3–1.78
	$\lambda =-0.0536{n}^{2} +0.6446n +0.3404$	N5	1.8–2.4	1.32–1.58
	$\lambda =0.1786{n}^{2} -0.6207n +1.8267$	N6	2–2.4	1.3–1.36

In a reflected type SPR, the incident light is reflected off the metal–dielectric interface, and only evanescent waves associated with plasmonic dipoles are excited. These evanescent waves decay rapidly away from the metal–dielectric interface, making them highly sensitive to changes in the refractive index of the analyte in contact with the metal surface. adding layers of materials such as silicon, graphene, and aGST can help increase the sensitivity of reflected type SPR by increasing the interaction between the evanescent wave and the analyte. Each layer added to the metal–dielectric interface can modify the properties of the evanescent wave, leading to changes in the SPR response. For example, adding a high refractive index layer such as aGST can shift the SPR angle to higher angles, making it more sensitive to changes in the refractive index of the analyte. Similarly, adding a graphene layer can enhance the electric field intensity at the metal–dielectric interface, leading to stronger SPR signals. From^70,71^ experimental data, the propagation length of SPP can be understood more clearly.

The following material interface effect can be identified for the generation of the resonance.

- *Prism-silicon interface* The prism-silicon interface is important for coupling the incident light into the structure and generating the evanescent wave that interacts with the SPP modes at the other interfaces. A high-index prism, such as a BK7 prism, can enhance the coupling efficiency and improve the sensitivity of the sensor.
- *Silicon-aGST interface* The silicon-aGST interface is important for supporting the SPP modes that are excited by the evanescent wave. The aGST layer can provide a high refractive index and a large thickness, which can increase the coupling efficiency and enhance the sensitivity of the sensor.
- *aGST-graphene interface* The aGST-graphene interface is important for supporting the plasmon modes in graphene, which can interact with the analyte layer and lead to changes in the reflectance spectrum. The plasmon modes in graphene can also be tuned by changing the doping level or by patterning the graphene with metal nanoparticles.
- *Graphene-silicon interface* The graphene-silicon interface is important for propagating the SPP wave that is generated at the silicon-aGST interface. The thickness and roughness of the graphene layer can affect the coupling efficiency and the propagation characteristics of the SPP wave.
- *Silicon-Ag grating interface* The silicon-Ag grating interface is important for coupling the SPP wave with the Ag grating and forming standing waves, which can enhance the sensitivity and resolution of the sensor. The spacing, depth, and shape of the Ag grating can affect the resonance conditions and the coupling efficiency of the SPP wave.
- *Ag grating-analyte interface* The Ag grating-analyte interface is important for interacting with the analyte molecules and leading to changes in the reflectance spectrum. The sensitivity and selectivity of the sensor depend on the specific properties of the analyte layer, such as its refractive index, thickness, and chemical composition.

## Influence of the different physical parameters of the proposed SPR sensor

**Figure 4:**
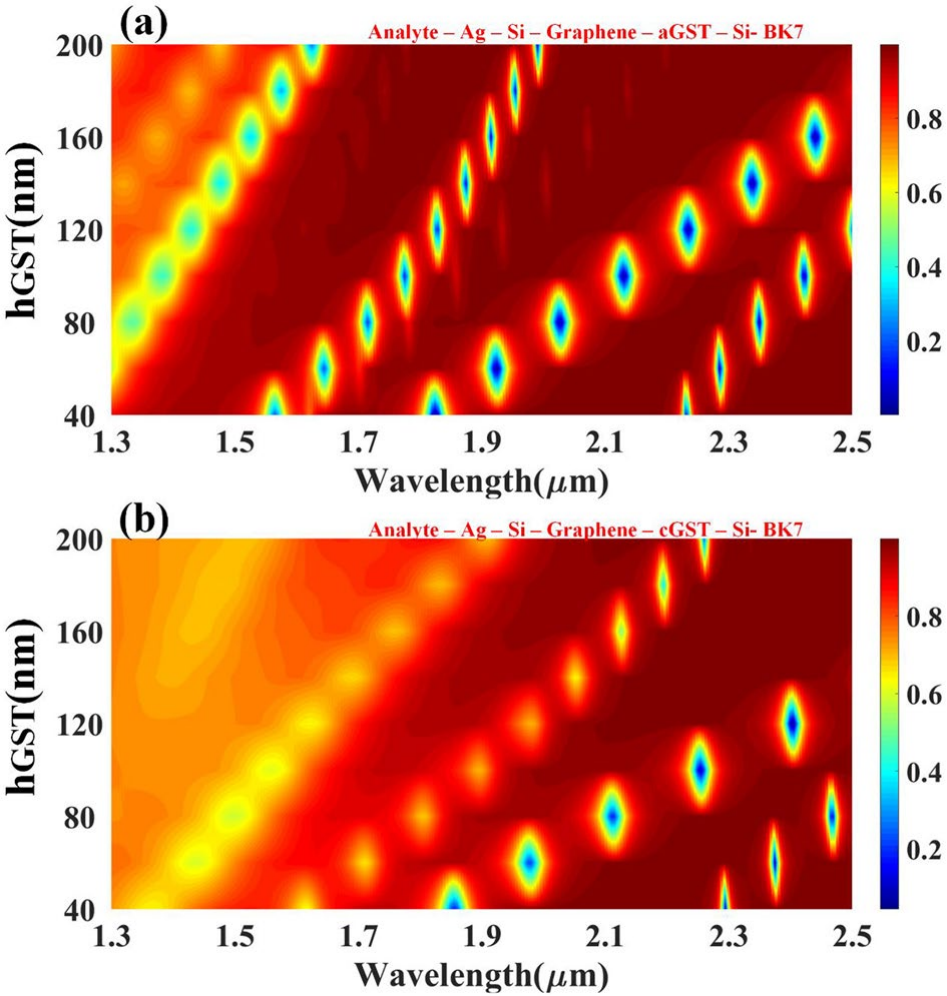
Contour map depicting the modification in resonating peaks for the multi-layered structure's simulated reflectance response as the wavelength function over the range of 1.3 to 2.5 µm w.r.t change in the height of the GST layer. Variation in the reflectivity distribution of the (**a**) aGST and (**b**) cGST phase of the material.

A crystalline and amorphous form of the GST material carries significant weight in detection, and the optimized value of the GST height goes a long way to detect changes in biological samples with greater precision by heightening sensitivity parameters. GST material height is varied from 40 to 200 nm to find the optimized value and to encounter the appropriate value of the GST height for the dedicated wavelength range to find the resonant shift. Figure 4 shows the impact of GST height on the reflectance response, with Fig. 4a showing aGST and Fig. 4b for cGST. For aGST, finer peaks were observed between 1.5 um to 2.5 um, whereas cGST is between 1.8 um to 2.5 um. Minimum reflectance is observed at a higher wavelength in both phases but better peaks were perceived in aGST in comparison with cGST because of the refractive index of the aGST phase. The resonance condition keeps on increasing by increasing the height of the GST without any changes in the reflectivity distribution for that particular wavelength range. The quadratic relationship has been established between the GST height and the resonance condition's wavelength. The solution of the quadratic equation will give you the optimized height of the GST for that precise wavelength range. The tunability of the structure can be easily spotted because of the obvious shift in the structures by changing the phases.

**Figure 5:**
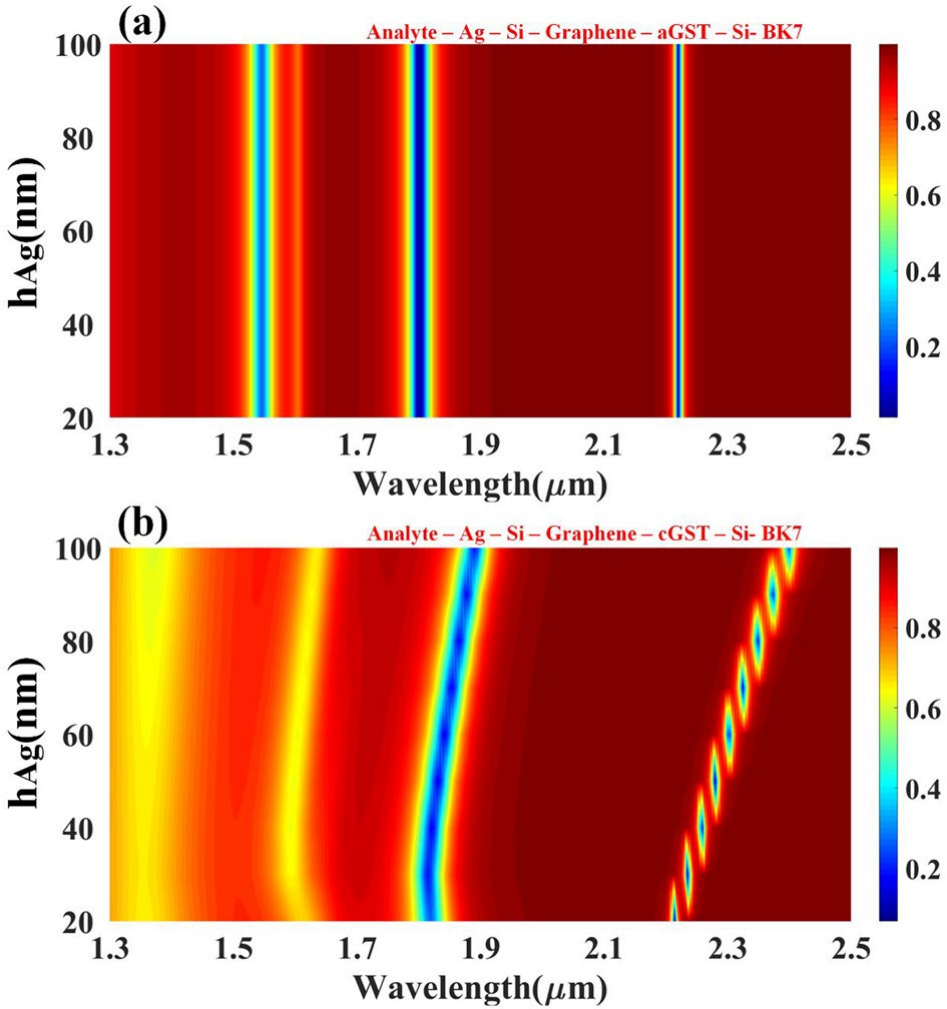
Calculated reflectance response for the different values of the silver resonator height for (**a**) aGST and (**b**) cGST phase of the proposed structure.

**Figure 6:**
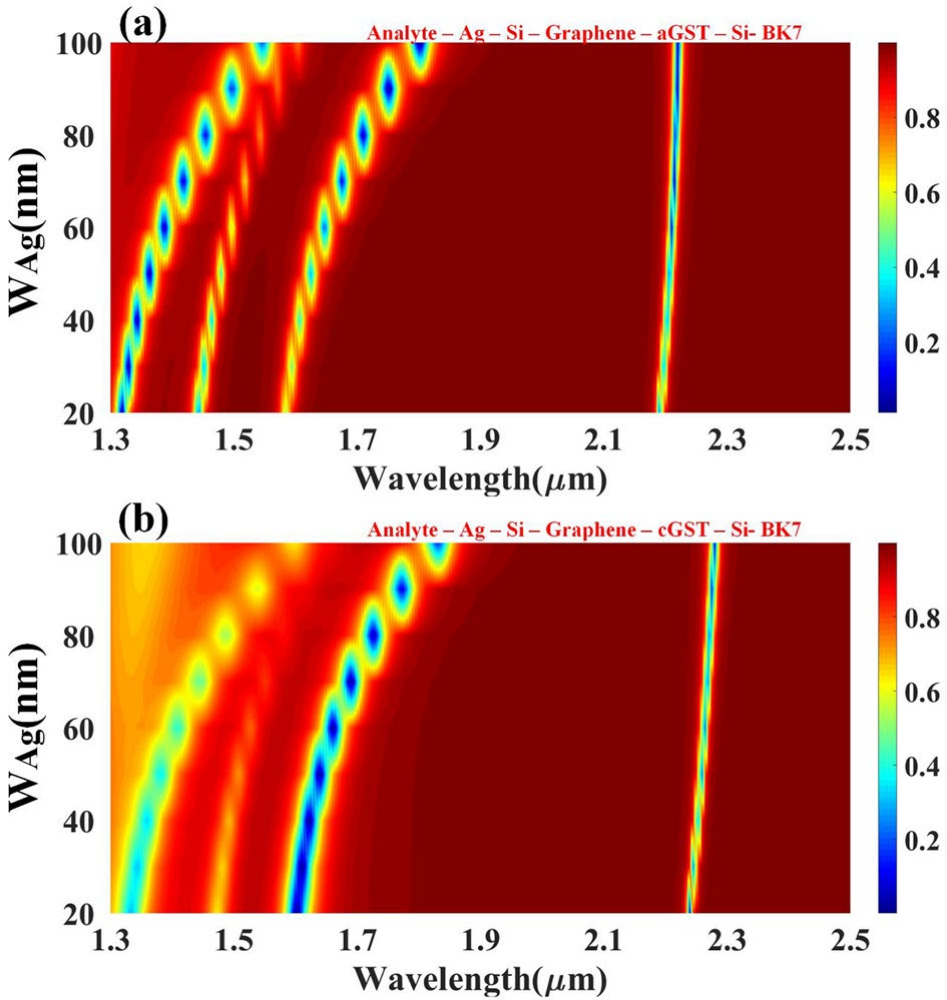
Calculated reflectance response for the different values of the silver resonator width for (**a**) aGST and (**b**) cGST phase of the proposed structure.

When used for sensing, the resultant SPR signal's breadth, location, and height must be very susceptible to variations in the refractive index of the dielectric substrate. In the proposed experimental setup, to a large extent, reflectance is affected by the experimental parameters, such as the metal film thickness in connection with the SPR sensor. Both the type of metal and its thickness greatly affect the plasmon curve's final form. This section analyses the impact of the height and width of the metal based on the reflectance values and resonant peaks achieved at the desired wavelength range. Figure 5 shows the impact of Ag height on the reflectance response with aGST and cGST phases of the material. Similarly, Fig. 6 shows the effect of Ag width on the material's reflectance response with aGST and cGST phases. From the Fig. 5, it can be clearly stated that for amorphous GST material, there is a linear relationship established between wavelength and height of the metal, while for crystalline GST material, there is a quadratic relation between metal height and wavelength, and precise results are obtained after 2.1 um and before that peaks are achieved with relatively maximum reflectance. Moreover, the impact of the width of the metals for both phases could be inferred by Fig. 6. Better reflectance curves are obtained for both phases between the 1.3 and 1.9 um wavelength range, and the tunability of the biosensor can be seen in the shift visible these two structures during whole wavelength range.

**Figure 7:**
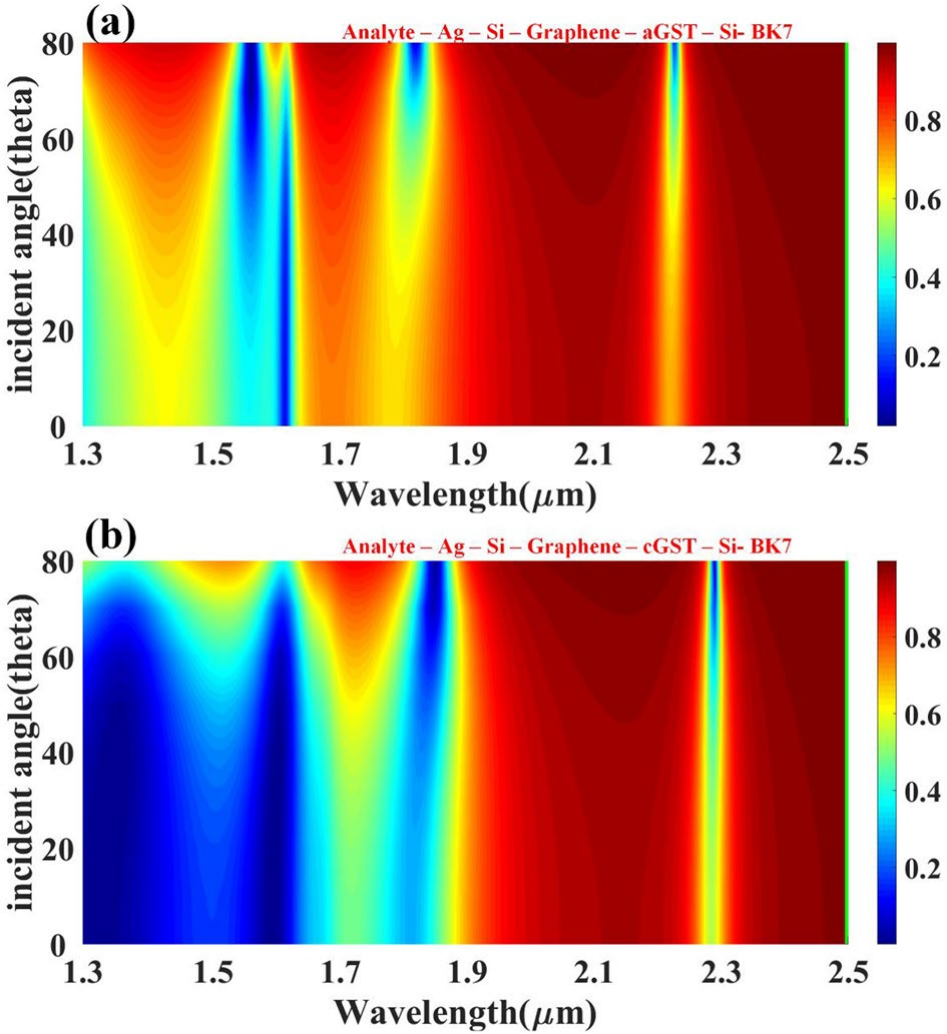
Aspects of reflectivity change to the incident angle of the input infrared wave for (**a**) aGST and (**b**) cGST phase of the material.

For the SPR excitation, the role of the incident angle is crucial. If the incidence angle were any larger or smaller, the resonance would not be formed, and the plasmonic effect would not occur. The metal film composition, the medium's refractive index, the incident light's wavelength, and ambient temperature all play a role in establishing the ideal resonant angle. Therefore, the impact of incident angle on finding the resonant peaks becomes the critical parameter. Figure 7 shows the effect of the angle of incidence on the reflectance response for the proposed structure. The incident angle varies with the analyte’s refractive index, but its optimum value can be found for a particular wavelength. For both forms, reflectance value changes are majorly seen between 1.3 and 1.9 um. In that region, minimum reflectance is achieved in cGST compared to aGST.

## Role of silver gratings in the proposed SPR biosensor and the choice of metal

**Figure 8:**
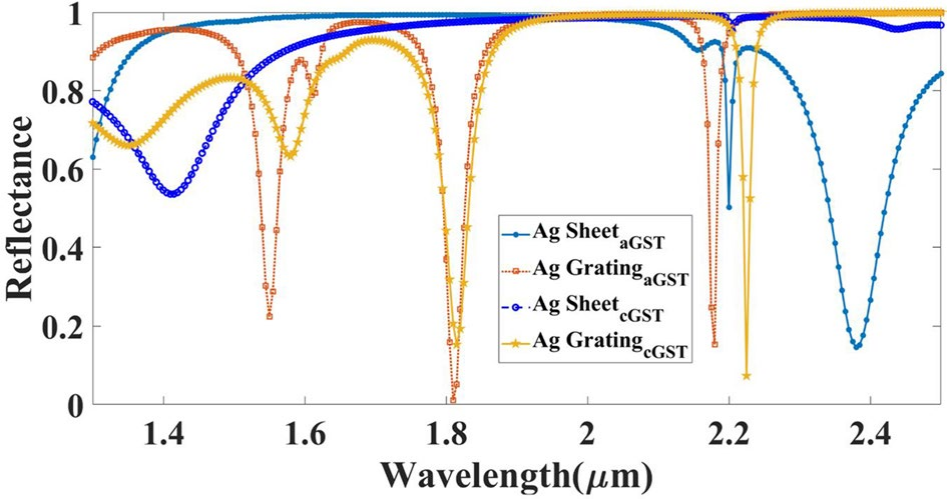
Calculated comparative reflectance response for the different types of the metal shape consideration such as flat sheet and grating.

Diffraction in a metal grating in a prism-based SPR biosensor can increase the electromagnetic field in the vicinity of the metal surface. The diffraction of light in the metal grating can enhance the sensitivity of the sensor by increasing the electromagnetic field in the vicinity of the metal surface. This enhanced field can increase the interaction between the target analyte and the biological layer on the metal surface, leading to a stronger signal and improved detection sensitivity. One of the major challenges in using thin silver films for biosensing in the infrared (IR) spectrum is that the plasmon resonance of thin silver films occurs at shorter wavelengths, typically in the visible or near-IR range. This limits their sensitivity and selectivity for biosensing in the IR region. One way to overcome this challenge is by using silver periodic nano-grating structures. These structures can support localized surface plasmon resonances (LSPRs) that can be tuned to the IR region by adjusting the grating period and depth. This allows for enhanced sensitivity and selectivity in IR biosensing applications. Figure 8 shows the comparative analysis of the grating-based structure and sheet-based structure. The proposed results clearly show the variation in the reflectance for the entire simulated wavelength range. In the normal sheet of the structure, we have observed the single resonance peak which can be tunable with a different phase of GST material. In grating-based design, it will observe the multiple resonance peaks over the entire wavelength spectrum.

**Figure 9:**
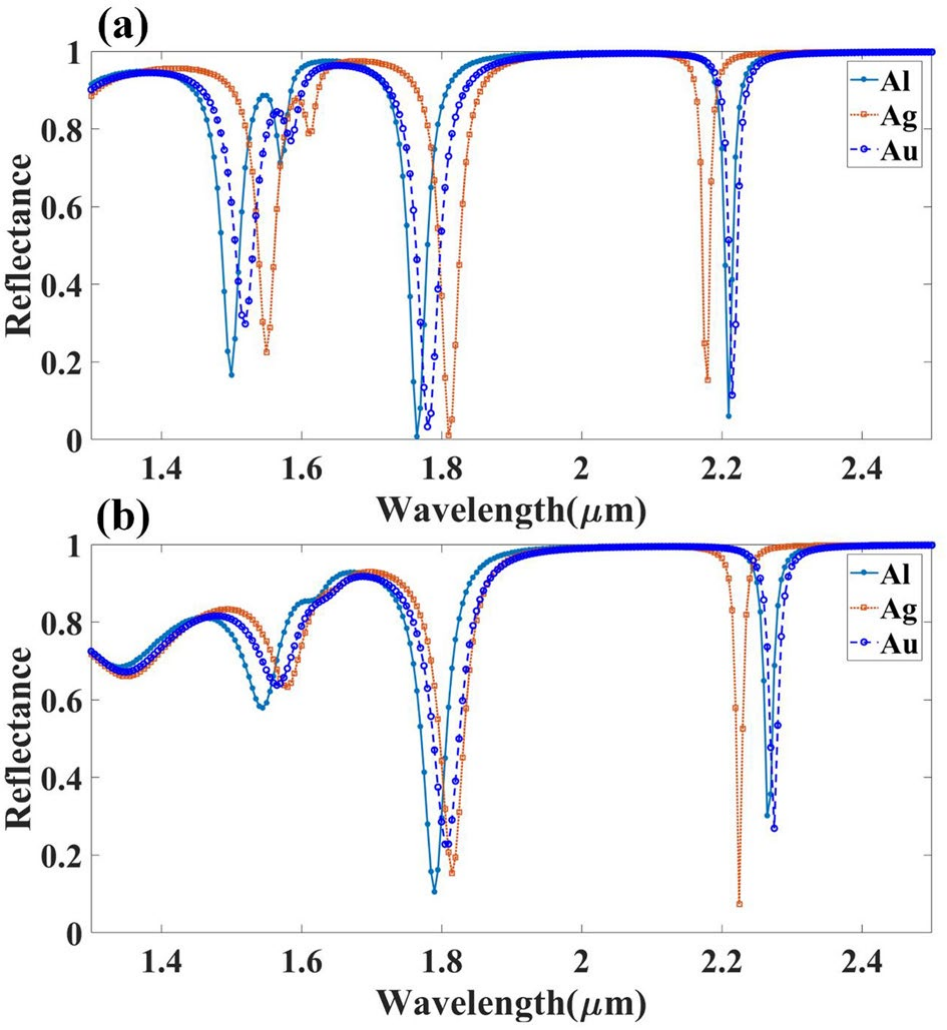
Calculated reflectance values for the different metallic materials chosen as the top grating layer (Al, Ag, and Au) for (**a**) aGST and (**b**) cGST phase of the material.

Moreover, the periodic nano-grating structures also have several advantages over thin silver films for IR biosensing. They can provide a larger surface area for biomolecule immobilization, which can enhance the sensitivity of the biosensor. They can also provide a reproducible and uniform surface for biomolecule immobilization, which can improve the reliability of the biosensor. Additionally, the LSPRs in the periodic nano-grating structures have narrow line widths, which can improve the selectivity of the biosensor^50,72^. In short, silver grating in the infrared region can help tailor to specific analyte detection providing better selectivity and sensitivity, which is seen in Fig. 8 with multiple resonance peaks. Additionally, the resonating performance also depends upon what type of metal you are choosing in grating but in the infrared region, the silver metal gives better plasmonic curves than gold because it has a higher plasmonic frequency, high absorption coefficient and better surface chemistry which gives strong plasmonic signals with better sensitivity to changes in the refractive index of the analyte. Aluminum is not preferred over silver because of its lower plasma frequency, higher optical losses, and surface chemistry limitations as well as oxidation issues. The variation in the reflectance for the different metallic materials of the grating structure (Aluminum-Al, Silver-Ag, and Gold-Au) is shown in Fig. 9. As a simulation results of the metallic part all are showing the results of the similar peaks generated for the entire spectrum but in sense of the oxidation and other surface chemistry, it can be recommended to use the Ag as resonating structure.

## Electrical distribution in the proposed SPR biosensor

**Figure 10:**
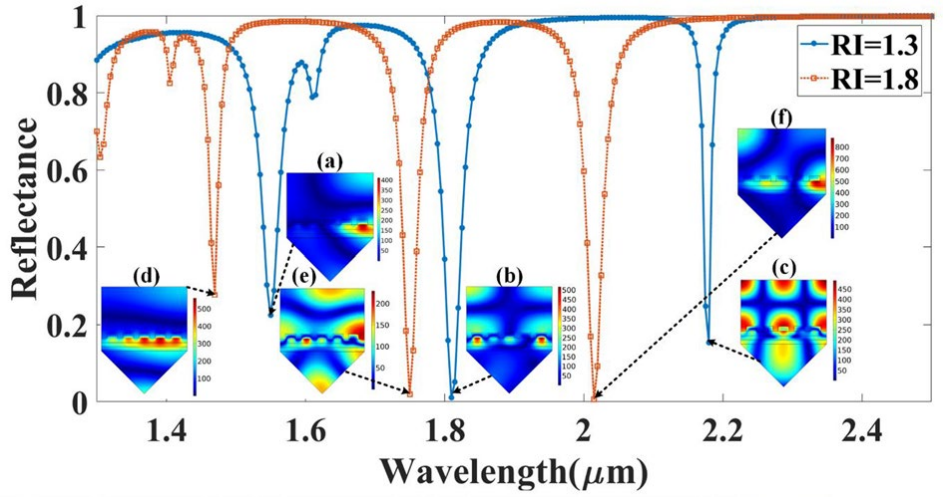
Distribution of the normalized electric field intensity for the different reflectance peaks for two refractive index values (RI = 1.3 and 1.8) with aGST phase of the GST material. The electric field distributions are presented at (**a**) λ = 1.55 µm, (**b**) λ = 1.81 µm, and (**c**) λ = 2.18 µm wavelength points for RI = 1.3 of the analyte. The electric field distributions are presented at (**d**) λ = 1.47 µm, (**e**) λ = 1.75 µm, and (**f**) λ = 2.01 µm wavelength points for RI = 1.8 of the analyte.

**Figure 11:**
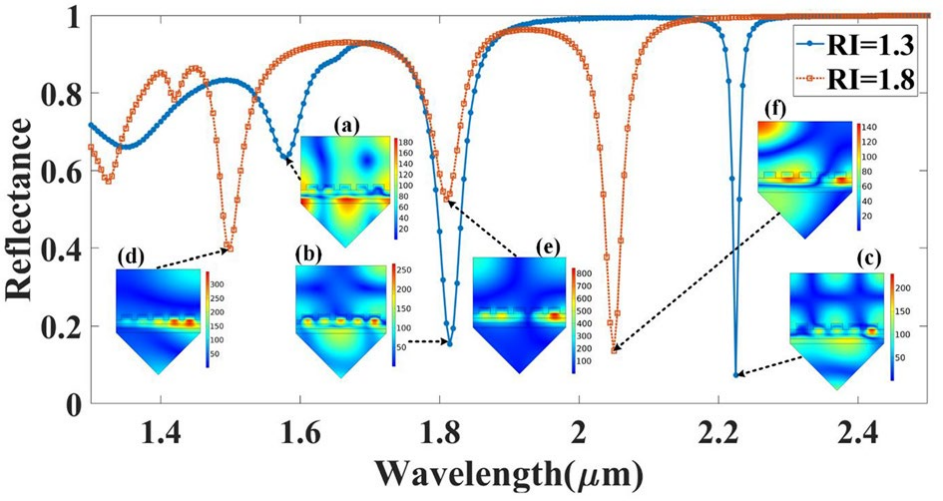
Distribution of the normalized electric field intensity for the different reflectance peaks for two refractive index values (RI = 1.3 and 1.8). The electric field distributions are presented at (**a**) λ = 1.58 µm, (**b**) λ = 1.81 µm, and (**c**) λ = 2.22 µm wavelength points for RI = 1.3 of the analyte with cGST phase of the GST material. The electric field distributions are presented at (**d**) λ = 1.5 µm, (**e**) λ = 1.81 µm, and (**f**) λ = 2.05 µm wavelength points for RI = 1.8 of the analyte.

At the interface between the silver (Ag) grating and the analyte layer, the SPR phenomenon occurs when the momentum of the incident light matches the momentum of the surface plasmon waves, leading to the collective oscillation of the free electrons in the metal layer. The electric field of the incident light is coupled with the electric field of the surface plasmon waves, leading to a strong enhancement of the electric field intensity at the metal–dielectric interface. This enhancement can lead to changes in the reflectance or transmission spectrum of the structure due to the interaction between the surface plasmon waves and the analyte layer. In the graphene layer, the localized plasmon modes can be excited by the incident light, leading to the formation of nanoscale electric field hotspots around the metal nanoparticles or patterns on the graphene layer. These hotspots are confined to the nanoscale regions and can interact with the analyte layer, leading to changes in the reflectance spectrum. The electrical distribution in the graphene layer is highly dependent on the doping level, patterning, and size of the metal nanoparticles or patterns. The electrical distribution in the other layers, such as the aGST layer, silicon layer, and prism, is also influenced by the presence of the surface plasmon resonances and localized plasmon modes in the adjacent layers. The coupling between the electric fields in different layers can lead to complex interference patterns and changes in the optical properties of the structure. Overall, the electrical distribution in the multilayer SPR structure is a complex interplay between the incident light, the surface plasmon resonances, and the localized plasmon modes in the various layers, and it depends on the specific details of the structure and the excitation conditions. Figure 10 and 11 depict the electrical distribution of various combinations of the proposed biosensor design for the different refractive index values of the analyte and different phases of the GST material.

Table 2 shows the detailed comparative analysis of the proposed multi-layered refractive index sensor with previously published designs regarding the type of structure, Materials, operating wavelength, refractive index range of sensing, and sensitivity. In this table, the sensitivity has been calculated using the following tracing curve $\lambda =0.1445{n}^{2}+ 0.0078n+1.5905$” which can be found in Table 1. Our proposed sensor provided approximately 2223 nm/RIU sensitivity for the broad wavelength range and refractive index values. In contrast, the other sensor offers high sensitivity values, but the operating range of wavelength and refractive index are limited in all comparison cases.

**Table 2:** Comparative analysis of the proposed refractive index sensor with previously published designs.

Type of the structure	Materials	Wavelength range	Refractive index range	Sensitivity	Ref
SPR-based multi-layered sensor for a wide range of refractive index sensing	Analyte-Ag-Silicon-Graphene-GST-Silicon-BK7	1.3–2.5 µm	1–2.4 with Δn = 0.1	~ 2223 nm/RIU	This design
V-shaped photonic crystal fiber with a high refractive index range	Gold -Silica	1.6 to 1.9	1.47–1.52	14,771 nm/RIU	^73^
D-shaped coated with graphene and zinc oxide	Graphene and Zinc Oxide	1.4–2.2	1.37 to 1.41	4485.7 nm/RIU	^74^
Graphene enhanced liquid refractive index sensor	Silica-Graphene_Gold	0.55- 0.75	1.3330–1.3688	2290 nm/RIU	^75^
PCF sensor with phase matching between the core mode and metal defect mode for a wide refractive index range	Fused Silica- Gold	1.2–1.8	1.34–1.46	1931.03 nm/RIU	^76^
Au nano wire-based optical fiber sensor	Gold-Glass	1.3–1.9	1.33–1.38	4471 nm/RIU	^77^
Graphene-Au coated SPR-based PCF	Graphene-Gold-Fused Silica	0.45–0.9	1.32–1.41	3900 nm/RIU	^78^
Highly sensitive PCF plasmonic biosensor	Fused silica–Gold	0.55–1.15	1.33–1.40	5142 nm/RIU	^79^
SPR biosensors using graphene and silicon layers	Graphene-Silicon-Gold	0.633	Δn = 0.005	89.7°/RIU	^64^
Graphene-based and Zinc Oxide assisted SPR biosensor	Graphene-Gold-Silver-Zinc Oxide	0.633	1.515	56.33°/RIU	^80^
Graphene-MoS2 with TiO2-SiO2 layers-based SPR	Graphene-MoS2-Au-SiO2-TiO2	0.633	1.34–1.41	81.33°/RIU	^81^
PCF filled with Gold nanowire encircled with Silicon filling	Fused Silica-Gold–Silicon	0.6–1.4	1.41–1.45	2666.67 nm/RIU	^82^
Grapefruit Fiber Filled with Silver Nanowires SPR	Fused Silica–Silver	0.6–0.7	1.330–1.335	2400 nm/RIU	^83^
Multi-layered–SPR sensor	MoS2–Gold–Silicon–prism	0.632	1.7786	53.49°/RIU	^41^

## Conclusion

This research provides the theoretical framework for a surface plasmon resonance-based biosensor with broad detection capabilities. By optimizing the height of GST material, silicon, silver, and the width of the silver, one may track the movement of the resonance dip of the reflected spectrum caused by modification of the incident light angle and the alteration of the refractive index. The performance of the proposed SPR-based generalized sensor has been computationally modeled and analyzed by following the Analyte–Ag–Si–Graphene–aGST–Si–BK7 or generally the sandwiched-based multilayer structure. The tunability of the sensor has been observed by simply adjusting the metal’s dimensions and changing the height of the silicon and GST. For a particular wavelength range, a generalized study of the optimized value of the parameters is given, which helps in SPR detection i.e. by fulfilling those specific conditions of wavelength and height and width of particular layers, several analytes with that detailed refractive index can be detected. Reflectance values results are comparatively best by adding the graphene layer. Numerous equations based on resonant traces have been provided to help researchers to calculate the sensing behavior at various wavelengths and refractive indices.

## Data Availability

Data are available based upon reasonable request from the corresponding author.
